# Supplementary material for: Effect of caesarean birth on perinatal mortality for singleton breech presentation in spontaneous preterm labour—A target trial emulation using Scottish health record data
Source: PLoS One. 2025 Jul 21;20(7):e0326001. doi: 10.1371/journal.pone.0326001 (PMC12279104; doi:10.1371/journal.pone.0326001)
Supplement: S1 Table — (DOCX) [file pone.0326001.s001.docx]

# TABLE S1: Defining the target trial and trial emulation.

|  | **Criteria** | **Target trial** | **Target trial emulation** |
| --- | --- | --- | --- |
| **Eligibility** | Inclusion/exclusion criteria using variables available at time of ‘enrolment’ | - Singleton pregnancy - Spontaneous labour onset - Preterm (24^+0^ to 36^+6^ weeks) - Breech presentation - No prior caesarean section | - Singleton pregnancy - Spontaneous labour onset - Preterm (24^+0^ to 36^+6^ weeks) - Breech presentation - No prior caesarean section |
| **Treatment strategy** | Specifically define treatment strategy, length of time participants must adhere to strategy, and valid reasons for derivation | Caesarean section vs. vaginal breech birth (unassisted or assisted) | Caesarean section vs. vaginal breech birth (unassisted or assisted) |
| **Outcomes** | Clearly define outcomes | *Modified* *extended perinatal death*, defined as the composite of intrapartum stillbirth and neonatal within 28 days of birth | *Modified* *extended perinatal death*, defined as the composite of intrapartum stillbirth and neonatal within 28 days of birth |
| **Treatment assignment** |  | Eligible women are randomly assigned to an intended mode of birth | Eligible women were classified according to final mode of birth.  We assumed that women were randomly assigned to mode of birth within the levels of the following variables:   - Maternal age - Maternal socioeconomic status - Maternal ethnicity - Marital status - Body mass index - Smoking status at antenatal booking, - Parity - Premature prelabour rupture of membranes - Previous stillbirth and/or neonatal death, - Gestational age - Presence of pre-existing maternal conditions - Occurrence of medical conditions arising during pregnancy - Occurrence of intrapartum complications - Existence of fetal conditions - Year of birth - Level of neonatal care available at the birth hospital |
| **Follow-up interval** | Define time zero and duration of follow-up. | - Eligibility and treatment assignment are determined at the time a woman presents in spontaneous labour. Birth occurs shortly after assignment. - Intrapartum survival is tracked from arrival at hospital to birth - Neonatal survival is tracked for the first 28 days following birth | - Eligibility and treatment assignment was determined using maternal, obstetric, and fetal characteristics known at the time of birth. Birth was assumed to occur shortly after assignment. - Intrapartum survival was estimated using birth and death records - Neonatal survival in the first 28 days following birth was determined using birth and death records |
| **Causal contrasts** |  | Per-protocol effect | Observational analogue of per-protocol effect |
| **Analysis** | Use of statistical techniques to adjust for baseline imbalance. | Per-protocol analysis with odds ratio as effect measure.  Gestation incorporated as effect modifier. | Per-protocol analysis with odds ratio as effect measure and gestation as effect modifier, augmented by inverse probability of treatment weights. |
