# Supplementary material for: Effect of caesarean birth on perinatal mortality for singleton breech presentation in spontaneous preterm labour—A target trial emulation using Scottish health record data
Source: PLoS One. 2025 Jul 21;20(7):e0326001. doi: 10.1371/journal.pone.0326001 (PMC12279104; doi:10.1371/journal.pone.0326001)
Supplement: S3 Table — (DOCX) [file pone.0326001.s003.docx]

# TABLE S3: Data sources

The target trial was emulated using routinely collected clinical data made available through the electronic Data Research and Innovation Service (eDRIS) of the National Health Service (NHS) Scotland. eDRIS curates and links sources of routinely collected data from within the NHS and other government bodies. Maternal records were matched using the community health index (CHI) number, a person-level identifier used across Scotland. Infant records were then matched to the mothers using either date of birth or date of death and child id (CHI). Data used within this study are summarised below.

| **Data Source** | **Dates** | **Subjects** | **Description** |
| --- | --- | --- | --- |
| Maternity hospital discharge records (SMR 02 database) | Jan 1997 – Dec 2019 | Mother/Pregnancy | Maternal pregnancy impatient and day case record |
| Scottish Stillbirth and Infant Death Survey (SSBID) | Jan 1997 – Dec 2012 | Mother/Pregnancy | Scottish Stillbirth and Infant Deaths Survey. Includes stillbirth, early neonatal deaths, and late neonatal deaths. |
| Mothers and Babies: Reducing Risk through Audits and Confidential Enquiries (MBRRACE) | Jan 2013 – Dec 2019 | Child | National surveillance investigation into deaths of women and babies who die during pregnancy or shortly after pregnancy. |
| National Records of Scotland (NRS) | Jan 1997 – Dec 2019 | Child | Contains national death records. |
| Scottish Morbidity Records for Neonates (SMR 11 database) | Jan 1997 – Jul 2003 | Child | Birth data for sick babies or babies with congenital anomalies. Healthy babies were recorded on SMR02 maternity record. Was replaced by SBR. |
| Scottish Birth Records (SBR database) | Mar 2002 – Dec 2019 | Child | Online system for every baby born in Scotland which includes all neonatal care in Scotland. Replaced SMR11. |
