# Supplementary material for: Effect of caesarean birth on perinatal mortality for singleton breech presentation in spontaneous preterm labour—A target trial emulation using Scottish health record data
Source: PLoS One. 2025 Jul 21;20(7):e0326001. doi: 10.1371/journal.pone.0326001 (PMC12279104; doi:10.1371/journal.pone.0326001)
Supplement: S4 Table — (DOCX) [file pone.0326001.s004.docx]

# TABLE S4. International Classification of Diseases (ICD) 10 codes for complications

| **Complication** | **ICD-10 Codes** |
| --- | --- |
| Hypertension | O10, O16 |
| Pre-eclampsia | O11, 014, O15 |
| Gestational hypertension | O13 |
| Pre-existing diabetes | O240, O241, O242, O243, O249 |
| Gestational diabetes | O244 |
| Liver disorder | O266 |
| Large-for-gestational-age fetus | O335, O336, O366 |
| Fetal abnormalities | O35, O360, O361, O362, O363, O430 |
| Intrauterine growth restriction | O365, O410 |
| Intrauterine infection | O411, O752, 0753 |
| Preterm prelabour rupture of membranes | O42 |
| Placenta accreta | O432 |
| Placenta previa | O44 |
| Placental abruption | O45 |
| Antepartum haemorrhage | O46 |
| Rupture of uterus | O710 |
| Chorioamnionitis | O20 |
