# Supplementary material for: Effect of caesarean birth on perinatal mortality for singleton breech presentation in spontaneous preterm labour—A target trial emulation using Scottish health record data
Source: PLoS One. 2025 Jul 21;20(7):e0326001. doi: 10.1371/journal.pone.0326001 (PMC12279104; doi:10.1371/journal.pone.0326001)
Supplement: S5 Table — (DOCX) [file pone.0326001.s005.docx]

# TABLE S5. Complication counts, by mode of birth.

| **Complication** | | | **Vaginal Birth**  N = 967 | **Caesarean Birth**  N = 2,092 | **Total**  N=3,059 |
| --- | --- | --- | --- | --- | --- |
|  | | | n (%) | n (%) | n (%) |
| Pre-existing hypertension | | |  |  |  |
|  | | No | 955 (98.2) | 2,060 (98.5) | 3,015 (98.6) |
|  | | Yes | 12 (1.2) | 32 (1.5) | 44 (1.4) |
| Pre-eclampsia | | |  |  |  |
|  | | No | 962 (99.5) | 2,025 (96.8) | 2,987 (97.7) |
|  | | Yes | 5 (0.5) | 67 (3.2) | 72 (2.3) |
| Gestational hypertension | | |  |  |  |
|  | | No | 962 (99.5) | 2,065 (98.7) | 3,027 (99.0) |
|  | | Yes | 5 (0.5) | 27 (1.3) | 32 (1.0) |
| Pre-existing diabetes | | |  |  |  |
|  | | No | 962 (99.5) | 2,063 (98.6) | 3,025 (98.9) |
|  | | Yes | 5 (0.5) | 29 (1.4) | 34 (1.1) |
| Gestational diabetes | | |  |  |  |
|  | | No | 957 (99.0) | 2,044 (97.7) | 3,001 (98.1) |
|  | | Yes | 10 (1.0) | 48 (2.3) | 58 (1.9) |
| Liver Disorders | | |  |  |  |
|  | | No | 962 (99.5) | 2,084 (99.6) | 3,046 (99.6) |
|  | | Yes | 5 (0.5) | 8 (0.4) | 13 (0.4) |
| Large-for-gestational-age fetus | | |  |  |  |
|  | | No | 962 (99.5) | 2,087 (99.8) | 3,049 (99.7) |
|  | | Yes | 5 (0.5) | 5 (0.2) | 10 (0.3) |
| Fetal abnormalities | | |  |  |  |
|  | | No | 952 (98.4) | 2,061 (98.5) | 3,013 (98.5) |
|  | | Yes | 15 (1.6) | 31 (1.5) | 46 (1.5) |
| Intrauterine growth restriction | | |  |  |  |
|  | | No | 911 (94.2) | 1,939 (92.7) | 2,850 (93.2) |
|  | | Yes | 56 (5.8) | 153 (7.3) | 209 (6.8) |
| Intrauterine infection | | |  |  |  |
|  | No | | 912 (94.4) | 2,000 (95.6) | 2,912 (95.2) |
|  | Yes | | 55 (5.7) | 92 (4.4) | 147 (4.8) |
| Placenta accreta | | |  |  |  |
|  | No | | 962 (99.5) | 2,087 (99.8) | 3,049 (99.7) |
|  | Yes | | 5 (0.5) | 5 (0.2) | 10 (0.3) |
| Placenta praevia | | |  |  |  |
|  | No | | 957 (99.0) | 2,054 (98.2) | 3,011 (98.4) |
|  | Yes | | 10 (1.0) | 38 (1.8) | 48 (1.6) |
| Placental abruption | | |  |  |  |
|  | No | | 941 (97.3) | 2,058 (98.4) | 2,999 (98.0) |
|  | Yes | | 26 (2.7) | 34 (1.6) | 60 (2.0) |
| Antepartum haemorrhage | | |  |  |  |
|  | No | | 899 (93.0) | 1,987 (95.0) | 2,886 (94.3) |
|  | Yes | | 68 (7.0) | 105 (5.0) | 173 (5.7) |
| Rupture of uterus | | |  |  |  |
|  | No | | 962 (99.5) | 2,087 (99.8) | 3,049 (99.7) |
|  | Yes | | 5 (0.5) | 5 (0.2) | 10 (0.3) |
| Chorioamnionitis | | |  |  |  |
|  | No | | 962 (99.5) | 2,087 (99.8%) | 3,049 (99.7) |
|  | Yes | | 5 (0.5) | 5 (0.2) | 10 (0.3) |
