# Supplementary material for: Effect of caesarean birth on perinatal mortality for singleton breech presentation in spontaneous preterm labour—A target trial emulation using Scottish health record data
Source: PLoS One. 2025 Jul 21;20(7):e0326001. doi: 10.1371/journal.pone.0326001 (PMC12279104; doi:10.1371/journal.pone.0326001)
Supplement: S6 Table — (DOCX) [file pone.0326001.s006.docx]

# TABLE S6. Characteristics of all singleton preterm breech births (including iatrogenic preterm births, previous caesarean birth) in Scotland between 1 January 1997 and 31 December 2019, by mode of birth.

| **Characteristic** | | **Vaginal Birth**  N = 1,318 | **Caesarean Birth**  N = 6,401 | **Total**  N = 7,719 |
| --- | --- | --- | --- | --- |
|  | | n (%) | n (%) | n (%) |
| Maternal age | |  |  |  |
|  | <20 | 139 (10.5) | 355 (5.5) | 494 (6.4) |
|  | 20-24 | 245 (18.6) | 916 (14.3) | 1,161 (15.0) |
|  | 25-29 | 336 (25.5) | 1,595 (24.9) | 1,931 (25.0) |
|  | 30-34 | 359 (27.2) | 1,872 (29.2) | 2,231 (28.9) |
|  | 35-39 | 195 (14.8) | 1,312 (20.5) | 1,507 (19.5) |
|  | ≥40 | 44 (3.3) | 351 (5.5) | 395 (5.1) |
| Marital Status | |  |  |  |
|  | Never Married | 445 (33.7) | 1,912 (29.9) | 2,357 (30.5) |
|  | Married | 345 (26.2) | 1,927 (30.1) | 2,272 (29.4) |
|  | Other/Unknown | 528 (40.1) | 2,562 (40.0) | 3,090 (40.0) |
| Ethnicity | |  |  |  |
|  | White | 421 (31.9) | 2,524 (39.3) | 2,945 (38.2) |
|  | Other | 31 (2.4) | 184 (2.9) | 215 (2.8) |
|  | Unknown/Refused | 866 (65.7) | 3,693 (57.5) | 4,559 (59.1) |
| SIMD | |  |  |  |
|  | 1 | 463 (35.2) | 1,804 (28.2) | 2,267 (29.4) |
|  | 2 | 308 (23.4) | 1,350 (21.1) | 1,658 (21.5) |
|  | 3 | 227 (17.3) | 1,130 (17.6) | 1,357 (17.6) |
|  | 4 | 161 (12.2) | 1,124 (17.5) | 1,285 (16.7) |
|  | 5 | 156 (11.9) | 977 (15.6) | 1,133 (14.7) |
| BMI | |  |  |  |
|  | Underweight | 27 (2.0) | 131 (2.0) | 158 (2.0) |
|  | Normal | 299 (22.7) | 1,543 (24.1) | 1,842 (23.9) |
|  | Overweight | 146 (11.1) | 919 (14.4) | 1,065 (13.8) |
|  | Obese | 127 (9.6) | 861 (13.5) | 988 (12.8) |
|  | Missing BMI | 719 (54.6) | 2,947 (46.0) | 3,666 (47.5) |
| Booking Smoking History | |  |  |  |
|  | Never | 574 (50.8) | 3,497 (61.1) | 4,071 (59.4) |
|  | Current | 451 (39.9) | 1,646 (28.7) | 2,097 (30.6) |
|  | Former | 105 (9.3) | 584 (10.2) | 689 (10.0) |
| Parity | |  |  |  |
|  | 0 | 550 (41.7) | 3,111 (48.6) | 3,661 (47.4) |
|  | 1 | 396 (30.0) | 1,748 (27.3) | 2,144 (27.8) |
|  | 2-4 | 328 (24.9) | 1,326 (20.7) | 1,654 (21.4) |
|  | 5+ | 32 (2.4) | 128 (2.0) | 160 (2.1) |
|  | Missing | 12 (0.9) | 88 (1.4) | 100 (1.3) |
| Gravidity | |  |  |  |
|  | 0 | 422 (32.0) | 2,310 (36.1) | 2,732 (35.4) |
|  | 1 | 349 (26.5) | 1,578 (24.7) | 1,927 (25.0) |
|  | 2 | 214 (16.2) | 1,037 (16.2) | 1,251 (16.2) |
|  | 3 | 129 (9.8) | 657 (10.3) | 786 (10.2) |
|  | 4 | 87 (6.6) | 337 (5.3) | 424 (5.5) |
|  | 5+ | 113 (8.6) | 457 (7.2) | 570 (7.4) |
| Previous spontaneous abortions | |  |  |  |
|  | No | 936 (71.1) | 4,445 (69.8) | 5,381 (70.0) |
|  | Yes | 381 (28.9) | 1,927 (30.2) | 2,308 (30.0) |
| Previous therapeutic abortions | |  |  |  |
|  | No | 1,172 (89.0) | 5,648 (88.6) | 6,820 (88.7) |
|  | Yes | 145 (11.0) | 724 (11.4) | 869 (11.3) |
| Previous neonatal death | |  |  |  |
|  | No | 1,290 (97.9) | 6,298 (98.7) | 7,588 (98.6) |
|  | Yes | 27 (2.1) | 82 (1.3) | 109 (1.4) |
| Previous Stillbirths | |  |  |  |
|  | No | 1,299 (98.6) | 6,202 (97.2) | 7,501 (97.5) |
|  | Yes | 18 (1.4) | 178 (2.8) | 196 (2.5) |
| Previous NND or SB | |  |  |  |
|  | No | 1,273 (96.7%) | 6,124 (96.0) | 7,397 (96.1) |
|  | Yes | 44 (3.3%) | 256 (4.0) | 300 (3.9) |
| Antenatal Steroids | |  |  |  |
|  | No | 233 (17.7) | 653 (10.2) | 886 (11.5) |
|  | Yes | 540 (41.0) | 2,811 (43.9) | 3,351 (43.4) |
|  | Not applicable | 125 (9.5) | 1,145 (17.9) | 1,270 (16.5) |
|  | Missing | 420 (31.9) | 1,792 (28.0) | 2,212 (28.7) |
| Sex of baby | |  |  |  |
|  | Female | 623 (47.3) | 3,179 (49.7) | 3,802 (49.3) |
|  | Male | 693 (52.7) | 3,219 (50.3) | 3,912 (50.7) |
| Gestational age at birth | |  |  |  |
|  | Extremely Preterm^2^ | 507 (38.5) | 449 (7.0) | 956 (12.4) |
|  | Very Preterm^2^ | 276 (20.9) | 1,253 (19.6) | 1,529 (19.8) |
|  | Moderately Preterm^2^ | 535 (40.6) | 4,699 (73.4) | 5,234 (67.8) |
| Level of neonatal care | |  |  |  |
|  | None | 59 (4.5) | 235 (3.7) | 294 (3.8) |
|  | LNU | 735 (55.8) | 3,835 (59.9) | 4,570 (59.2) |
|  | SCU | 74 (5.6) | 434 (6.8) | 508 (6.6) |
|  | NICU | 450 (34.1) | 1,897 (29.6) | 2,347 (30.4) |
| PPROM | |  |  |  |
| Yes | | 1,056 (80.1) | 5,218 (81.5) | 6,274 (81.3) |
| No | | 262 (19.9) | 1,183 (18.5) | 1,445 (18.7) |
| Complications^1^ | |  |  |  |
| No | | 964 (73.1) | 3,899 (60.9) | 4,863 (63.0) |
| Yes | | 354 (26.9) | 2,502 (39.1) | 2,856 (37.0) |
|  | |  |  |  |
| Birth outcomes | |  |  |  |
| Extended perinatal death | | 276 (20.9) | 221 (3.5) | 497 (6.4) |
| Stillbirth | | 62 (4.7) | 10 (0.2) | 72 (0.9) |
| Early neonatal death (0-6 days) | | 174 (13.2) | 143 (2.2) | 317 (4.1) |
| Late neonatal death (7-28 days) | | 40 (3.0) | 68 (1.1) | 108 (1.4) |

Data are n (%).

BMI, body mass index; LNU, local neonatal unit; NICU, neonatal intensive care unit; PPROM, preterm prelabour rupture of membranes; SCU, special care unit; SMID, Scottish Index of Multiple Deprivation.

^1^ Complications, defined as the presence at least one of the following conditions: pre-existing or gestational hypertension, pre-eclampsia, pre-existing or gestational diabetes, liver disorders, large-for-gestational age fetus, fetal abnormalities, intrauterine growth restriction, infection, chorioamnionitis, placenta accreta, placenta praevia, placental abruption, antepartum haemorrhage, or rupture of the uterus

^2^ Extremely preterm was defined as 24^+0 to^ 27^+6^ gestational weeks; very preterm as 28^+0^ to 31^+6^ gestational weeks; and moderately preterm as 32^+0^-36^+6^ gestational weeks.
