# Supplementary material for: Effect of caesarean birth on perinatal mortality for singleton breech presentation in spontaneous preterm labour—A target trial emulation using Scottish health record data
Source: PLoS One. 2025 Jul 21;20(7):e0326001. doi: 10.1371/journal.pone.0326001 (PMC12279104; doi:10.1371/journal.pone.0326001)
Supplement: S7 Table — (DOCX) [file pone.0326001.s007.docx]

# TABLE S7. Odds ratios and numbers needed to treat, by gestational week.

| **Weeks of gestation** | **Odds Ratio** | **95% confidence interval** | **Numbers needed to treat** |
| --- | --- | --- | --- |
| 24 weeks | 0.526 | 0.353 to 0.784 | 7 |
| 25 weeks | 0.481 | 0.342 to 0.677 | 7 |
| 26 weeks | 0.440 | 0.328 to 0.589 | 8 |
| 27 weeks | 0.402 | 0.312 to 0.518 | 7 |
| 28 weeks | 0.368 | 0.291 to 0.464 | 16 |
| 29 weeks | 0.336 | 0.266 to 0.424 | 15 |
| 30 weeks | 0.307 | 0.238 to 0.396 | 27 |
| 31 weeks | 0.281 | 0.210 to 0.376 | 70 |
| 32 weeks | 0.257 | 0.182 to 0.361 | 15 |
| 33 weeks | 0.235 | 0.158 to 0.349 | 24 |
| 34 weeks | 0.214 | 0.136 to 0.339 | 17 |
| 35 weeks | 0.196 | 0.116 to 0.330 | 26 |
| 36 weeks | 0.179 | 0.100 to 0.323 | 88 |
