# Supplementary material for: Effect of caesarean birth on perinatal mortality for singleton breech presentation in spontaneous preterm labour—A target trial emulation using Scottish health record data
Source: PLoS One. 2025 Jul 21;20(7):e0326001. doi: 10.1371/journal.pone.0326001 (PMC12279104; doi:10.1371/journal.pone.0326001)
Supplement: S1 Text — (DOCX) [file pone.0326001.s008.docx]

# TEXT S1: Participant identification

**Approach used to define spontaneous onset of labour**

The four logic pathways used to identify women in spontaneous labour were:

- Women with premature rupture of membranes
- Women with no premature rupture of membranes that had no recorded induction of labour with a vaginal birth
- Women with no premature rupture of membranes that had no recorded induction of labour and laboured more than 1 hour but did not ultimately birth vaginally
- Women with no premature rupture of membranes that had no recorded induction of labour and an unknown length of labour resulting in an emergency caesarean birth

**Approach used to define antepartum stillbirths**

Stillbirths were classified as antepartum (death prior to labour onset) or intrapartum (death after onset of labour and prior to birth) using the ‘age at death’ indicator within the SSBID database and the indicator for live fetus at the onset of care from the MBRRACE database. Intrapartum stillbirths were retained in the trial cohort.
